# Nicotinic acetylcholine receptor-mediated effects of varenicline on LPS-elevated prostaglandin and cyclooxygenase levels in RAW 264.7 macrophages

**DOI:** 10.3389/fmolb.2024.1392689

**Published:** 2024-05-27

**Authors:** Elif Baris, Mualla Aylin Arici, Metiner Tosun

**Affiliations:** ^1^ Department of Medical Pharmacology, Faculty of Medicine, Izmir University of Economics, Izmir, Türkiye; ^2^ Department of Medical Pharmacology, Faculty of Medicine, Dokuz Eylul University, İzmir, Türkiye

**Keywords:** varenicline, α7nAChR, inflammation, cyclooxygenase, prostaglandins

## Abstract

**Introduction:** The purpose of this study is to delineate anti-inflammatory and antioxidant potential of varenicline, a cigarette smoking cessation aid, on decreasing lipopolysaccharide (LPS)-elevated proinflammatory cytokines in RAW 264.7 murine macrophage cultures which we showed earlier to occur via cholinergic anti-inflammatory pathway (CAP) activation. To this end, we investigated the possible suppressive capacity of varenicline on LPS-regulated cyclooxygenase (COX-1 and COX-2) via α7 nicotinic acetylcholine receptor (α7nAChR) activation using the same *in vitro* model.

**Materials and Methods:** In order to test anti-inflammatory effectiveness of varenicline, the levels of COX isoforms and products (PGE2, 6-keto PGF1α, a stable analog of PGI2, and TXA2) altered after LPS administration were determined by Enzyme Linked Immunosorbent Assay (ELISA). The antioxidant effects of varenicline were assessed by measuring reductions in reactive oxygen species (ROS) using a f*luorometric* intracellular *ROS *a*ssay kit*. We further investigated the contribution of nAChR subtypes by using non-selective and/or selective α7nAChR antagonists. The results were compared with that of conventional anti-inflammatory medications, such as ibuprofen, celecoxib and dexamethasone.

**Results:** Varenicline significantly reduced LPS-induced COX-1, COX-2 and prostaglandin levels and ROS to an extent similar to that observed with anti-inflammatory agents used.

**Discussion:** Significant downregulation in LPS-induced COX isoforms and associated decreases in PGE2, 6-keto PGF1α, and TXA2 levels along with reduction in ROS may be partly mediated via varenicline-activated α7nAChRs.

## 1 Introduction

Lipopolysaccharide (LPS), an endotoxin from Gram-negative bacteria, induces “pathogen-associated molecular pattern (PAMP) recognition receptors” (toll-like receptors, TLRs) expressed on immune system cells. Exposure to LPS initiates an inflammatory response that includes unrestrained production of pro-inflammatory cytokines; tumor necrosis factor (TNF), interleukins (IL-1, IL-6, and IL-8), platelet-activating factor (PAF) from immune cells. Reactive Oxygen Species (ROS) formed due to various factors cause the oxidant and antioxidant balance in cells to be disrupted and the tissue to develop an inflammatory reaction due to these factors (Palsson-McDermott and O'Neill, 2004; Murdock and Núñez, 2016).

The release of pro-inflammatory cytokines can set off a systemic inflammatory response, can be regulated by the cholinergic system, which is pivotal in regulating the body’s reaction to inflammation and ensuring survival. This regulatory process is facilitated by the cholinergic anti-inflammatory pathway (CAP), a sophisticated neural network that diminishes the release of pro-inflammatory cytokines through the vagus nerve and the stimulation of nicotinic receptors found on a variety of immune cells, including lymphocytes, macrophages, and others (Fujii et al., 2017; Snider et al., 2018). Pharmacological activation of cholinergic receptors and electrical stimulation of the vagus nerve have proven effective in reducing cytokine production in a range of conditions such as ischemia and sepsis. Clinical studies have highlighted that therapies targeting the CAP can significantly improve organ function and reduce mortality rates in sepsis patients (Zimmermann et al., 2017; Pinder et al., 2019a; Pinder et al., 2019b). A significant amount of research has concentrated on pharmacological means to activate cholinergic receptors, especially the α7nAChR on immune cells, which are instrumental in the interaction between the cholinergic and immune systems. The inhibition of inflammatory mediators like TNFα, IL-6, and IL-8 by α7nAChR agonists, including substances like nicotine and GTS-21, is noteworthy. These agonists also modulate the response to pro-inflammatory stimuli, like LPS-induced activation of cyclooxygenase (COX) and subsequent prostaglandin (PG) production, underscoring a significant anti-inflammatory role. Moreover, these agents not only regulate cytokine levels but also enhance survival rates across various experimental models by dampening the inflammatory response (Pavlov et al., 2007; Fujii et al., 2017; Zimmermann et al., 2017; Snider et al., 2018). Hence, agents that activate the α7nAChRs are increasingly being recognized for their potential therapeutic value in managing inflammation, offering a promising avenue for treatment strategies (Bernik et al., 2002; Zimmermann et al., 2017; Pinder et al., 2019a; Pinder et al., 2019b).

Pro-inflammatory cytokines and the direct effect of endotoxin also increase the expression of the COX isoform COX-2, which plays a significant role in inflammation. While the constitutive form COX-1 is mostly associated with homeostasis, COX-2 plays significant functions in inflammation and carcinogenesis, and is activated by cytokines, tissue damage, and tumor promoters. PGI_2_ and PGE_2_ are also involved in elevation of body temperature, one of the fundamental signs of inflammation. PGE_2_ and PGI_2_ induce vasodilation, increasing blood flow, and mediate leukocyte infiltration, pain, and edema. PGI_2_ is not stored and is rapidly converted to its inactive metabolite 6-keto-PGF1α. Thromboxane A2 (TXA2) is a potent vasoconstrictor and platelet aggregator that plays a crucial role in hemostasis and thrombosis. Studies have shown that LPS can upregulate COX-2 expression in various cell types, including macrophages and endothelial cells leading to elevation of TXA2, which contributes to the pro-inflammatory and pro-thrombotic effects. In addition, LPS was shown to stimulate PGD2 production through the activation of the nuclear factor-kappa B (NF-κB) signaling pathway involved in the regulation of allergic and inflammatory responses (Ricciotti and FitzGerald, 2011; Joo and Sadikot, 2012). The release and circulation of PGs and pro-inflammatory cytokines generate an inflammatory response, leading to an increase in capillary permeability, hemodynamic changes, extensive endothelial cell damage, septic shock, sepsis, and multiple organ failure (Bernik et al., 2002; Baris et al., 2021a; Bigagli et al., 2021).

In our previous study, varenicline decreased LPS-induced inflammatory cytokine levels in RAW 264.7 murine macrophage cell lines without significant difference with dexamethasone. Furthermore, varenicline significantly reduced LPS-induced cell migration through α7nAChR, while decreasing cell proliferation independently of nAChR. Our findings suggested that varenicline attenuates LPS-induced inflammation by activating α7nAChRs, eventually reducing cytokine production and cell migration (Baris et al., 2021b). Moreover, CAP-inducing agents; CDP-choline and choline, reduce the inflammation process through the COX pathway in LPS-induced endotoxemia in rats. Following LPS administration, COX-2 expressions and PG levels increased, both of which were significantly reduced by CDP-choline or choline treatment via α7nAChRs (Baris et al., 2021a; Baris et al., 2023a), highlighting the potential of α7nAChRs to mediate modulation of the COX pathway.

Varenicline, which is widely used as an effective and safe therapeutic option for smoking cessation, is reported to have potent and full agonistic properties on α7nAChRs and partial agonistic effects on α4β2-nAChRs (Baris et al., 2021b; Bigagli et al., 2021). A recent clinical study showed that 12-week varenicline treatment modulated inflammation and oxidative damage (Baris et al., 2023a). Furthermore, immunohistochemical experiments showed that varenicline treatment suppressed inflammation and the number of immune system cells through α7nAChR activation in brain and lung tissues in an animal model of ischemia and emphysema (Mihalak et al., 2006a; Hays et al., 2008). However, varenicline’s effects on LPS-induced COX pathway and oxidative stress development are not known. Therefore, this study investigated varenicline’ α7nAChR-mediated effects on COX, PG and ROS levels in LPS-exposed RAW 264.7 murine macrophages.

## 2 Materials and methods

### 2.1 Cell culture

RAW 264.7 murine macrophage cells at passage #8 (ATCC TIB-71, Manassas, VA) were maintained in DMEM (Sigma Aldrich D6429), supplemented with heat-inactivated FBS (10%) and penicillin (100 U/mL), streptomycin (100 μg/mL, Gibco, Carlsbad, CA) at 37°C in 5% CO_2_ incubator. Regular checks for *mycoplasma* contamination were performed with a *mycoplasma* detection kit (Biowest, Riverside, MO). Cells (500,000/well) were seeded in 48-well tissue culture plates after detachment with scraping incubated for 24 h in serum-free media for reattachment to the surface. Before adding chemicals, the medium was replaced with fresh serum-free media (DMEM supplemented with penicillin-streptomycin without FBS) for all treatment groups. In the first group, cells were treated with LPS (*Escherichia coli*, Sigma Aldrich L4130 0111: B4) at various concentrations (1–2 and 3 μg/mL) to determine effective concentration at which cytokines are released (Parrish et al., 2008). In the second group, cells were pretreated with varenicline tartrate (Sigma-Aldrich PZ0004) with increasing concentrations (0.1-0.3-0.8-1-3-10 μM) 30 min prior to LPS administration to determine effective varenicline concentration on LPS-induced COX and PG levels. The non-cytotoxic concentrations of varenicline were determined earlier in our study (Baris et al., 2021b). Additionally, the effect of varenicline was compared with that of ibuprofen (0.5 µM, Santa Cruz sc-200534), celecoxib (3 μM, MedChem HY-14398) and dexamethasone (0.1 μΜ, Sigma Aldrich D4902) (Jeon et al., 2000; Bigagli et al., 2021). In the third group, to investigate the involvement of nicotinic receptors, a non-selective nAChR antagonist mecamylamine hydrochloride (MEC, 50 μΜ, Sigma Aldrich M9020) and selective α7nAChR antagonist methyllycaconitine citrate (MLA, 1 μΜ, Sigma Aldrich M168) were applied 30 min before varenicline and LPS (Yang et al., 2015; Yi et al., 2015; Baris et al., 2021b). RAW 264.7 cells at passage #5 were originally from ATTC (gift). *Mycoplasma* contaminations were performed with a *mycoplasma* detection kit (Biowest, Riverside, MO).

### 2.2 Protein and ROS analyses

The levels of COX-1 (BT-Lab E0955Mo), COX-2 (Elabscience M0959), PGE_2_ (Elabscience-E-EL-0034), 6-keto PGF1α (Elabscience E-EL-0054) and TXA2 (Elabscience E-EL-0057) levels released into the culture media 24 h after LPS administration were determined by Enzyme-Linked Immunosorbent Assay (ELISA) according to the manufacturer’s instructions. Reactive oxygen species (ROS) were measured via a fluorimetric ROS kit (Elabscience, E-BC-K138-F) according to manufacturer’s guidelines.

### 2.3 Statistical analysis

The Shapiro-Wilk test was employed to analyze normal data distribution. One-way analysis of variance analysis (ANOVA) with *post hoc* Tukey-Kramer multiple comparison tests or Student’s *t*-test (GraphPad Prism 5, La Jolla, CA) were used to compare means to compare means of data distributed parametrically. Data were expressed as mean *±* standard error of the mean (SEM) (n = 6, each performed in triplicate) and *p <* 0.05 was accepted as statistically significant.

## 3 Results

### 3.1 LPS-elevated COX and PG levels

**FIGURE 1:**
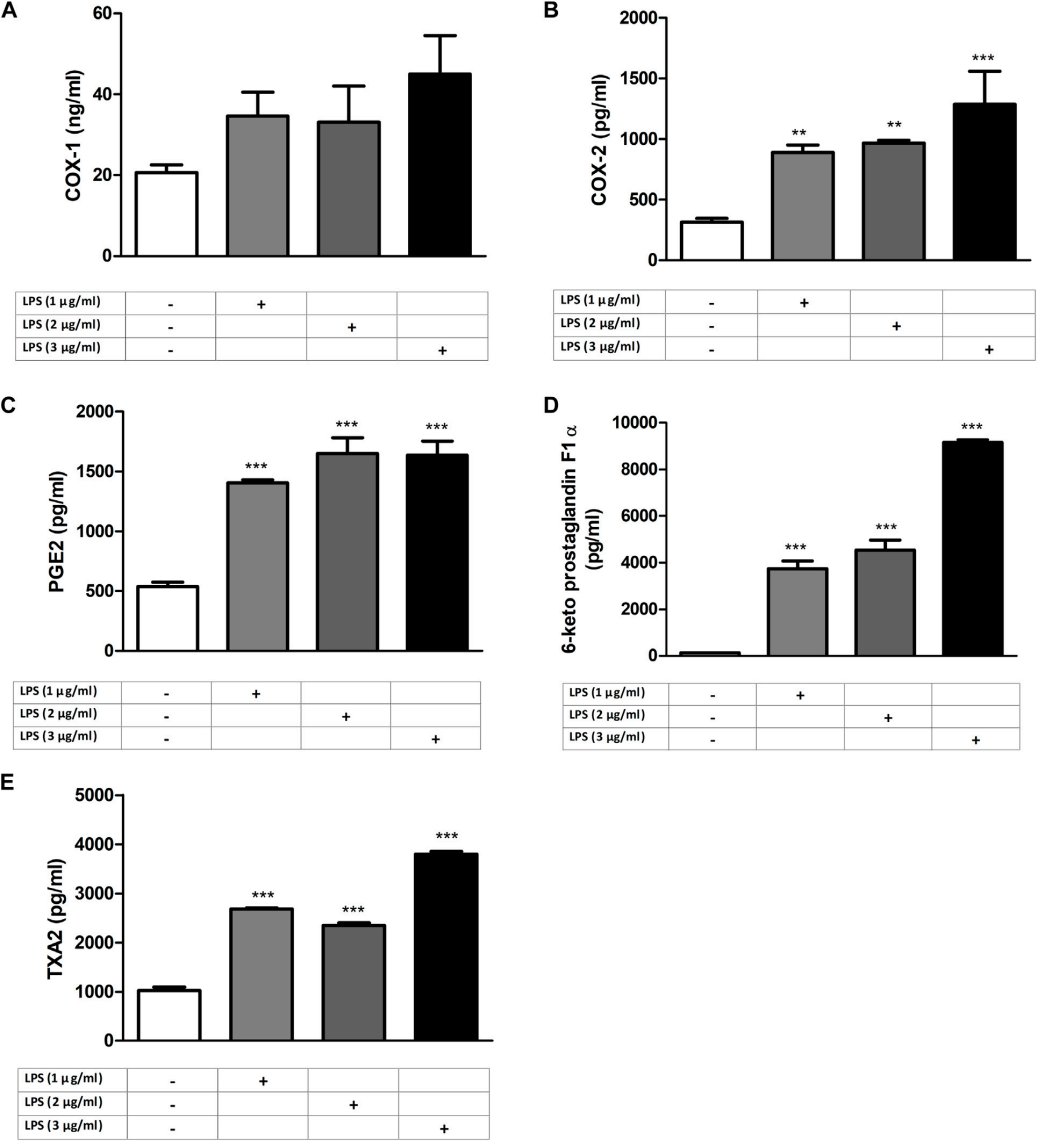
LPS-induced increase in COX and PG levels in RAW264.7 cells. Shown are COX-1 **(A)**; COX-2 **(B)**, PGE2 **(C)**, 6-keto PGF1α **(D)**, and TXA2 **(E)** levels in response to increasing LPS concentrations. Data are shown as mean *±* S.E.M. (**, *p <* 0.01; ***, *p <* 0.001 vs. control, n = 6, and One-way ANOVA with *post hoc* Tukey-Kramer multiple comparison test or Student’s *t*-test). LPS: Lipopolysaccharide, COX: Cyclooxygenase, PG: Prostaglandin, TXA2: Thromboxane A2.

RAW 264.7 cells were exposed to increasing concentrations of LPS (1–2 and 3 μg/mL) for 24 h before analyzing COX and PG levels to determine the effective concentration of LPS to be used. COX-2 and PG levels were LPS increased by LPS in a concentration-dependent manner (*p* < 0.001, n = 6) comparable to that of control and the 1 μg/mL LPS group (Figure 1).

### 3.2 Inhibitory effects of varenicline on LPS-elevated COX and PG levels

**FIGURE 2:**
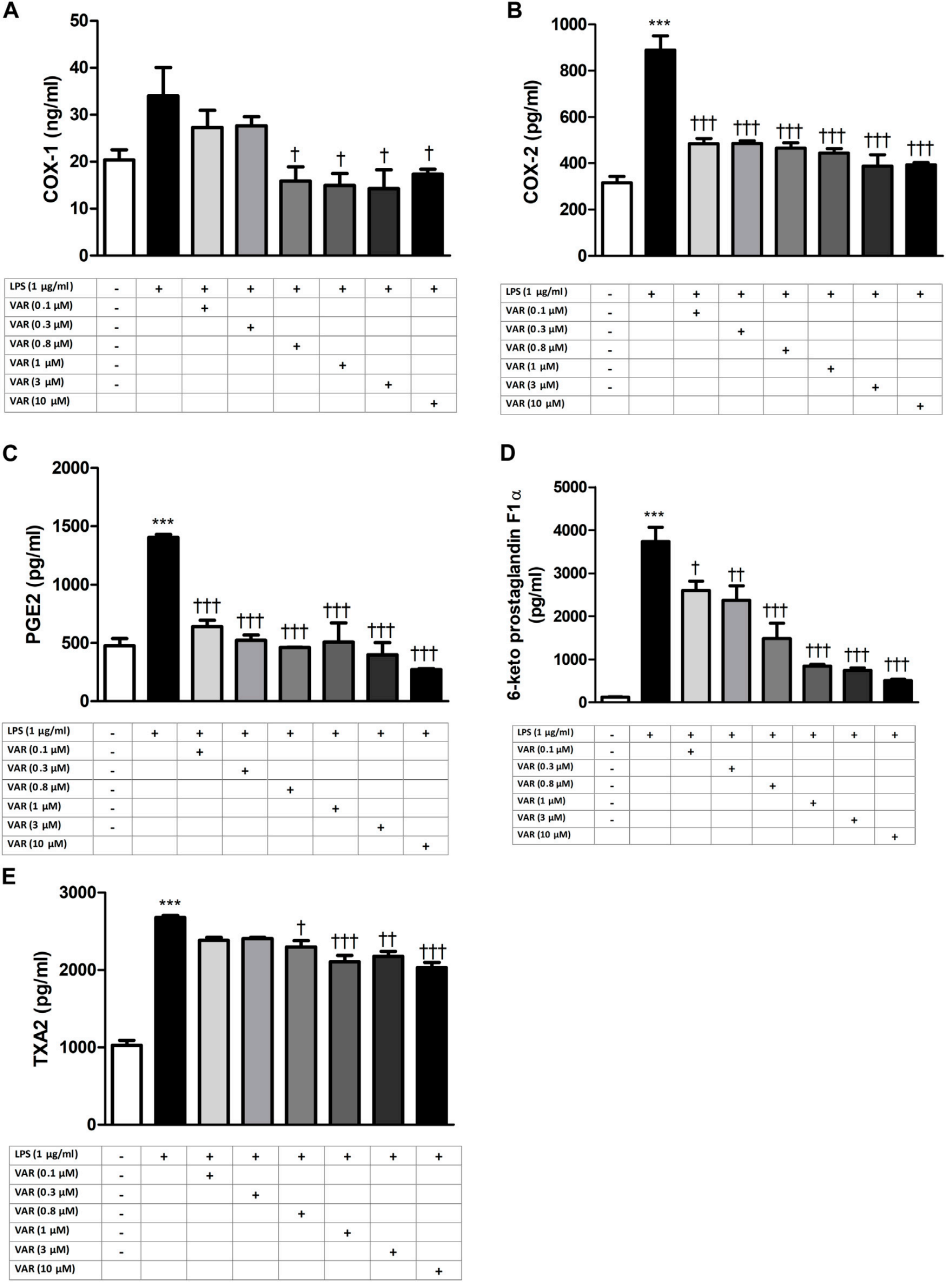
Effects of varenicline on LPS-induced COX and PG elevations. Shown are the effects of varenicline on 1 μg/mL LPS -induced COX-1 **(A)**; COX-2 **(B)**, PGE2 **(C)**, 6-keto PGF1α **(D)** and TXA2 **(E)** levels. Data are shown as mean ± S.E.M. (***, *p* < 0.001, LPS vs. control; ^†^, *p <* 0.05, ^††^, *p* < 0.01, ^†††^, *p <* 0.001, VAR vs. LPS, n = 6, One-way ANOVA with *post hoc* Tukey-Kramer multiple comparison test or Student’s *t*-test). LPS: Lipopolysaccharide, VAR: Varenicline, PGE2: Prostaglandin E2, TXA2: Thromboxane A2.

**FIGURE 3:**
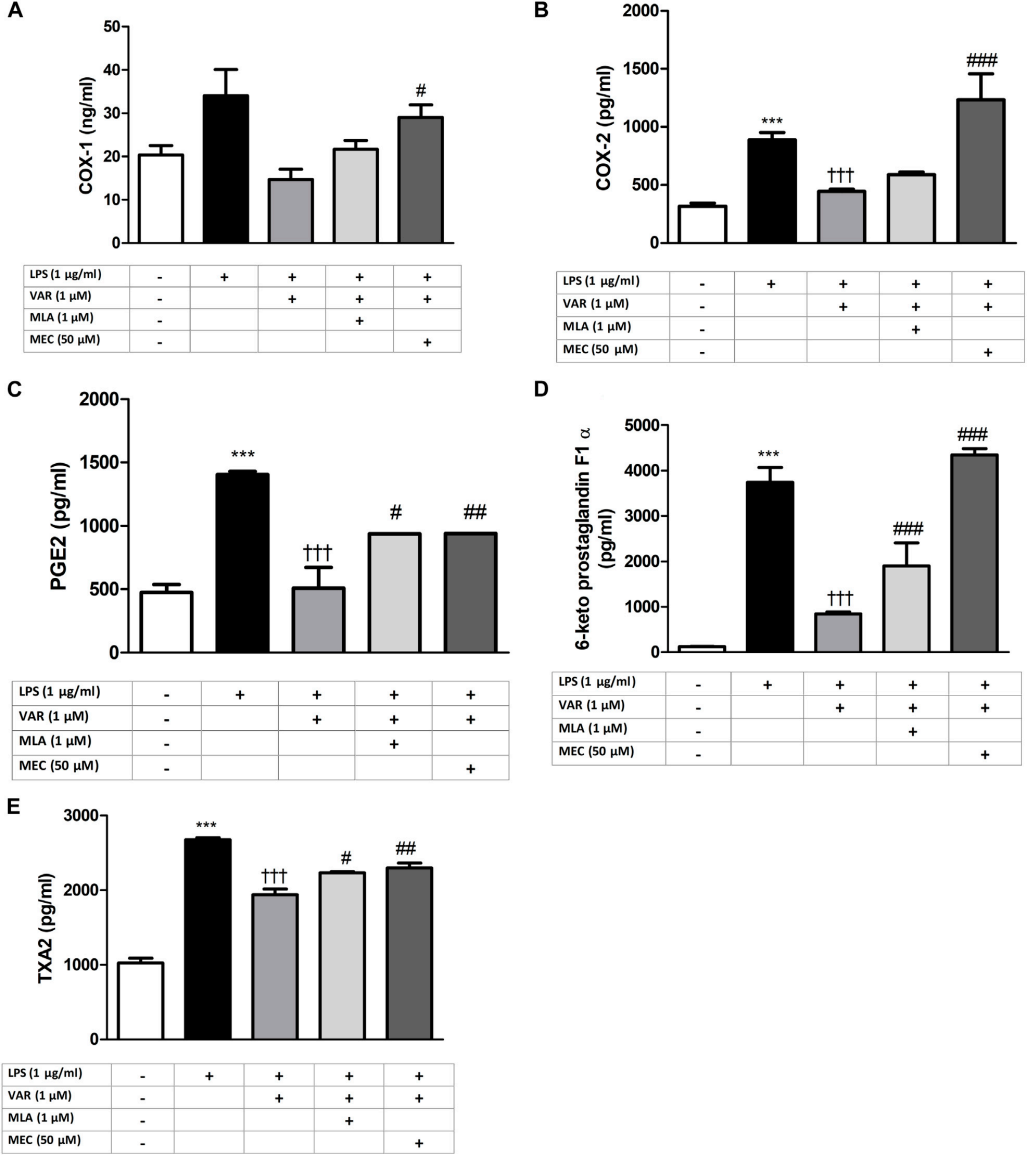
Effects of varenicline on LPS-induced COX and PG elevations in the presence or absence of nAChR antagonists. Shown are 1 μg/mL LPS-elevated COX-1 **(A)**; COX-2 **(B)**, PGE2 **(C)**, 6-keto PGF1α **(D)** and TXA2 **(E)** levels in the absence or presence of varenicline (VAR, 1 μΜ), mecamylamine (MEC, 50 μΜ) and methyllycaconitine (MLA, 1 μΜ). Data are shown as mean ± S.E.M. (***, *p* < 0.001 vs. control, ^†††^, *p <* 0.001 vs. LPS; ^‡^, *p* < 0.05, ^‡‡^, *p* < 0.01, ^‡‡‡^, *p* < 0.001 vs. LPS + VAR, n = 6, One-way ANOVA with *post hoc* Tukey-Kramer multiple comparison test or Student’s *t-*test). LPS: Lipopolysaccharide, VAR: Varenicline, MLA: Methylylcaconitine citrate, MEC: Mecamylamine. COX: Cyclooxygenase, PGE2: Prostaglandin E2, TXA2: Thromboxane A2.

RAW 264.7 cells were pretreated with increasing concentrations of varenicline (0.1–0.3-0.8-1-3-10 μΜ) 24 h prior to administration of predetermined LPS concentration (1 μg/mL). Varenicline suppressed LPS-elevated COX and PG levels (Figure 2). Higher concentrations of varenicline (>1 µM) did not further inhibit PG and COX levels. LPS-elevated COX and PGs were also suppressed by ibuprofen, celecoxib and dexamethasone; however, the data did not reach a statistical significancy (Figure 3). Levels of these parameters (PG, IL-6 and TNFα) were not altered by drug treatment and DMSO *per se* (not shown).

### 3.3 nAChR-mediated suppression of LPS-elevated COX, PG and ROS levels by varenicline

**FIGURE 4:**
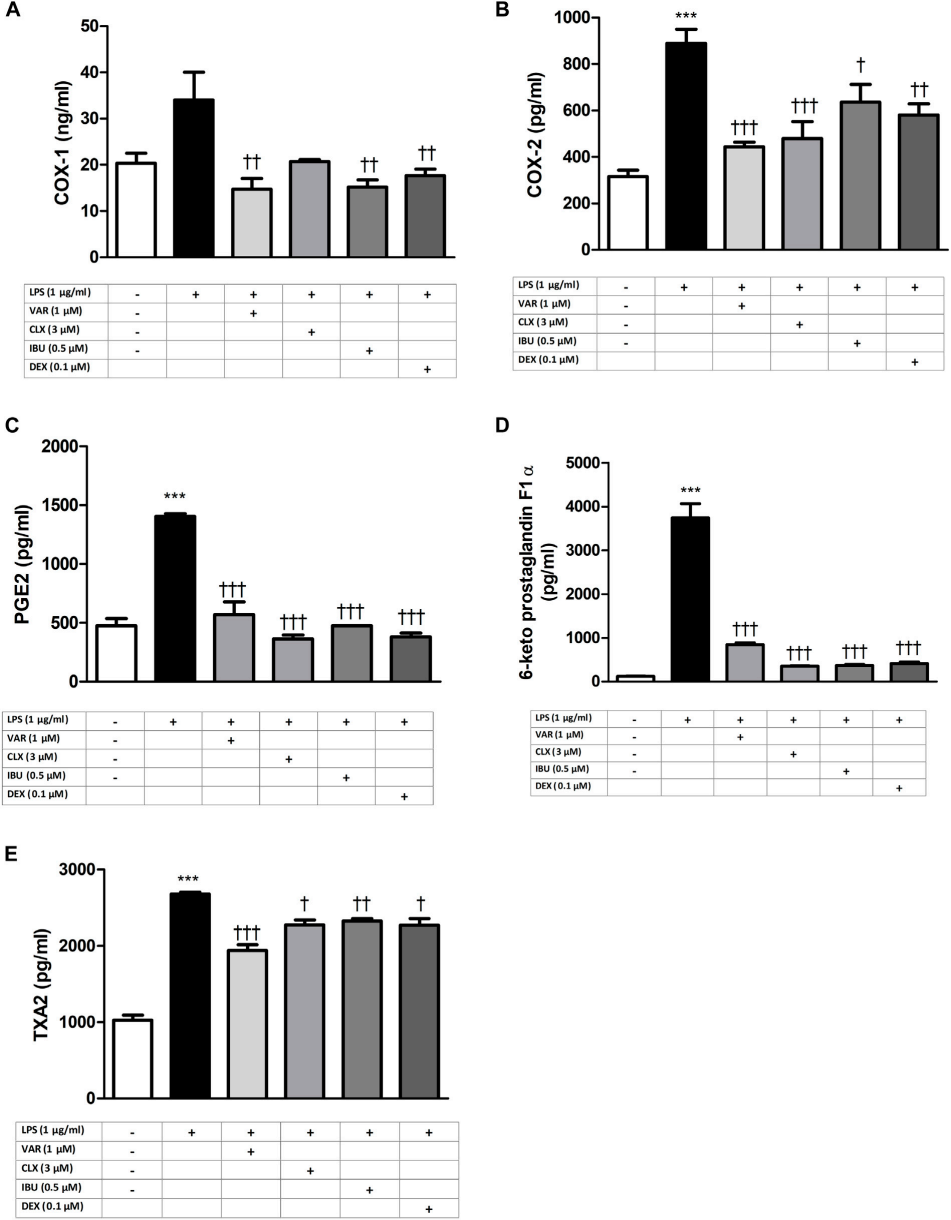
Effects of varenicline and conventional anti-inflammatory agents on LPS-induced COX and PG elevations. Shown are the effects of varenicline on 1 μg/mL LPS -induced COX-1 **(A)**; COX-2 **(B)**, PGE2 **(C)**, 6-keto PGF1α **(D)**, TXA2 **(E)** levels and the comparison with celecoxib, ibuprofen and dexamethasone. Data are shown as mean ± S.E.M. (***, *p* < 0.001 vs. control; ^†^, *p <* 0.05; ^††^, *p* < 0.01, ^†††^, *p <* 0.001 vs. LPS, n = 6, One-way ANOVA with *post hoc* Tukey-Kramer multiple comparison test or Student’s *t*-test). LPS: Lipopolysaccharide, VAR: Varenicline, CLX: Celecoxib, IBU: Ibuprofen, DEX: Dexamethasone. COX: Cyclooxygenase, PGE2: Prostaglandin E2, TXA2: Thromboxane A2.

**FIGURE 5:**
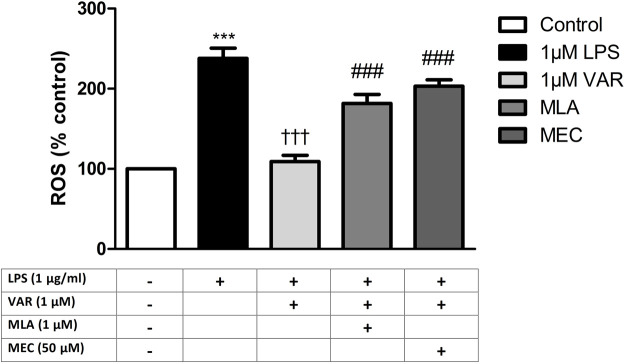
Effects of varenicline on LPS-induced ROS elevations in the presence or absence of nAChR antagonists. Shown are 1 μg/mL LPS-elevated ROS levels in the absence or presence of varenicline (VAR, 1 μΜ), mecamylamine (MEC, 50 μΜ) and methyllycaconitine (MLA, 1 μΜ). Data are shown as mean ± S.E.M. (***, *p* < 0.001 vs. control, ^†††^, *p <* 0.001 vs. LPS; ^‡^, *p* < 0.05, ^‡‡^, *p* < 0.01, ^‡‡‡^, *p* < 0.001 vs. LPS + VAR, n = 6, One-way ANOVA with *post hoc* Tukey-Kramer multiple comparison test or Student’s *t-*test). LPS: Lipopolysaccharide, VAR: Varenicline, MLA: Methylylcaconitine citrate, MEC: Mecamylamine.

RAW 264.7 cells were pretreated with mecamylamine (MEC) and/or methyllycaconitine citrate (MLA) prior to the incubation with varenicline (1 μΜ) and LPS (1 μg/mL) for 24 h. COX, PG and ROS levels significantly increased in MEC and MLA groups compared to varenicline-treated groups (Figures 4, 5).

## 4 Discussion

This study shows that varenicline significantly inhibits LPS-induced COX-1 and COX-2 elevations via α7nAChR activation. Decreases in PGE2, PGI_2_, PGI_2_ metabolite 6-keto PGF_1α_ and TXA2 along with ROS levels, suggest varenicline’s potential value in prevention of COX-mediated and oxidative response through CAP activation.

LPS is known to initiate inflammation by promoting the production of cytokines and PGs (Parrish et al., 2008; Yang et al., 2015; Yi et al., 2015; Temiz-Resitoglu et al., 2017; Baris et al., 2021a). Arachidonic acid metabolites synthesized by COX are PGE_2_ cause swelling, edema at the site of infection or tissue damage and TXA2 activates platelet aggregation repetitively during tissue injury and inflammation. COX-2 is an inducible enzyme expressed in response to inflammatory stimuli. Therefore, in the first part of this study, RAW 264.7 macrophages, commonly used in LPS-induced inflammation models, were treated with increasing concentrations of LPS to determine optimal LPS concentration (1 μg/mL) to induce inflammation. The increase in the levels of PGs and COX that mediate inflammation confirms the development of inflammation, consistent with the earlier observations (Gandhi et al., 2015; Baris et al., 2023a). Varenicline, used as a reliable option for smoking cessation, exhibits strong agonistic activity on α7nAChRs (Baris et al., 2021b; Bigagli et al., 2021). The distinction in PG's roles emphasizes the scope and direction of our study, while acknowledging the comprehensive landscape of prostaglandin function in various physiological and pathophysiological processes, including but not limited to inflammation, immune responses and homeostasis. Only a few studies reports its potential anti-inflammatory effects in different models (Mihalak et al., 2006a; Hays et al., 2008; Baris et al., 2023a). Varenicline has been shown to decrease inflammation in lung tissue of animals with emphysema (Hays et al., 2008) and reduce brain inflammation in animals with stroke (Mihalak et al., 2006a). A clinical study has also demonstrated that varenicline significantly decreased eicosanoid-related inflammation and oxidative damage in patients during smoking cessation therapy (Baris et al., 2023a). Consistent with these, our previous study showed that varenicline decreased LPS-induced inflammatory cytokine levels in RAW 264.7 macrophage cells (Baris et al., 2021b). It is known that glucocorticoids are potent anti-inflammatory agents that modulate inflammation response through the attenuation of cytokine release whereas non-steroidal anti-inflammatory drugs (NSAIDs) (i.e., ibuprofen or a selective COX-2 inhibitory medication, celecoxib) decrease PG levels by inhibiting COX (Yeboah et al., 2008; Yui et al., 2015). These drugs have been shown to decrease LPS-induced cytokine and PG release in RAW 264.7 cells (Jeon et al., 2000; Kondreddy and Kamatham, 2016; Ai et al., 2020; Won et al., 2021). Our results showed no significant difference between varenicline and dexamethasone or ibuprofen and celecoxib regarding the inhibitory effects on COX expression and PG elevation. Therefore, regardless of mechanism of action and efficacy, varenicline’s anti-inflammatory properties did not differ statistically when compared with dexamethasone or NSAIDs which needs to be confirmed with additional studies.

Nicotinic AChRs play important roles in the development of pain and inflammation associated with inflammatory pain models (Koga et al., 2018). The increase in COX and PG levels in the presence of nonselective and selective nAChR antagonists, mecamylamine (MEC) and/or methyllycaconitine citrate (MLA), suggests α7nAChR involvement in anti-inflammatory effects of varenicline. Anti-inflammatory effectiveness of varenicline in lung and brain tissues in mouse models of emphysema and stroke models has been shown to be mediated by α7nAChR activation (Joo and Sadikot, 2012; Baris et al., 2021a). These studies provide indirect evidence for the anti-inflammatory role of varenicline without investigating its effects on inflammatory cytokine levels. Accumulating evidence suggest that α7nAChRs expressed on immune cells are required to balance the endogenous response to inflammation through activation of cholinergic system (Pinder et al., 2019a). Agents acting on α7nAChRs have been shown to inhibit LPS-induced inflammatory response in various *in vivo* and *in vitro* studies (Pavlov et al., 2003; Wang et al., 2003; Pavlov et al., 2007; Hays et al., 2008; Parrish et al., 2008; Chen et al., 2017; McElroy et al., 2018). Several molecular mechanisms have been suggested for the α7nAChR-mediated inhibition of pro-inflammatory cytokines in macrophages such as inhibiting the nuclear translocation of transcription factor NF-κB and JAK2/STAT3 signaling pathway (De Jonge and Ulloa, 2007; Bagdas et al., 2015; Kondreddy and Kamatham, 2016). Our data provide experimental evidence by showing an α7nAChR agonist varenicline suppresses PG synthesis/release through a receptor-dependent mechanism and CAP. However, downstream intracellular mechanisms were not investigated in the present study. The effects of varenicline on COX pathway may also be potentiated by decreases in LPS-elevated cytokine levels (Mühl and Dinarello, 1997; Li et al., 2014; Baris et al., 2021b).

Inflammation and oxidative stress are intricately linked to the development of inflammatory response. It has been shown that the expression levels of cytokines and COX-2 are elevated in endotoxemic animals. Concurrently, levels of malondialdehyde (MDA) and hydrogen peroxide (H_2_O_2_), alongside cytokine concentrations, increased, while the levels of catalase and glutathione decreased in the brain tissues of these mice (Sirijariyawat et al., 2019). Moreover, α7nAChR expression ACh levels, and choline acetyltransferase activity also decreased (Han et al., 2018). We previously demonstrated the therapeutic efficacy of CAP-inducing agents, choline and CDP-choline, in mitigating LPS-induced elevations in ROS, TNFα, and NF-κB levels (Baris et al., 2023b). Studies performed on the effects of a 3-month smoking cessation program using varenicline on vascular function and oxidative stress markers showed that after 3 months, participants have decreased levels of carbon monoxide (CO), MDA, protein carbonyls (PC), and augmentation index (Aix), indicating reduced arterial stiffness and oxidative stress. In addition, another study assessing urinary biomarkers like PGE2 metabolite (PGE-M) and 8-iso-PGF2α revealed that smoking cessation for 84 days significantly decreased these markers, reflecting a reduction in systemic inflammation and oxidative damage. These findings indicate varenicline’s potential value in mitigating inflammation and oxidative stress in individuals who quit smoking (Ikonomidis et al., 2017; McElroy et al., 2018). Consistent with the clinical findings, our data support the nAChR-mediated effectiveness of varenicline on LPS-induced ROS and COX upregulation.

Varenicline, primarily known for its use in smoking cessation, has a unique mechanism of action as a partial agonist at nicotinic acetylcholine receptors. While its common side effects include nausea, headaches, and changes in dreaming, its cholinergic effects, akin to those observed with cholinergic drugs, might present differently due to its selective receptor activity. Cholinergic side effects, such as increased salivation, sweating, and gastrointestinal disturbances, could potentially arise from varenicline’s action but are not as prominently documented or understood (Baker et al., 2021).

## 5 Limitations

Exploring the intracellular dynamics of varenicline’s impact on inflammation and other prostaglandins i.e., PGD2 and PGF2α were not within the objectives of this study. While the pre-clinical findings seem significant, further preclinical *in vivo* and clinical research are necessary to validate the suppressive action of varenicline on the LPS-induced COX pathway through α7nAChR activation.

## 6 Conclusion

It is known that PGs, produced via COX in arachidonic acid pathway, mediate the cardinal signs of inflammation, including pain, increased body temperature, redness, edema, and loss of function. Today, corticosteroids and nonsteroidal anti-inflammatory drugs (NSAIDs), used for pain and inflammation control, are the most frequently used agents in the treatment of inflammatory diseases due to their effects on the COX pathway. Despite their gastrointestinal, hematological, cardiovascular, and hepatotoxic side effects, there is no alternative medication to NSAIDs, which are widely used clinically. Selective inhibitors of COX-2, a subtype of COX expressed mainly in macrophages and functions in the inflammatory pathway, are known to lead to an increased incidence of severe cardiovascular events. Apart from that, novel non-steroidal anti-inflammatory drugs (NSAIDs) via COX-2 aim to reduce cardiovascular risks associated with traditional NSAIDs. Several compounds starting from lumiracoxib were developed, having dual COX-2 inhibitory activity on COX-2 and TP receptors within the same molecule. However, none of them yielded optimal results for therapeutic use (Hoxha et al., 2016). Although lumiracoxib failed to gain FDA approval due to hepatotoxicity risks, it has paved the way for the development of novel safer coxibs, emphasizing the need for alternative NSAIDs for pain management in patients with high cardiovascular risks. In our *in vitro* inflammation model conducted in rodent macrophage cell lines, the anti-inflammatory efficacy of varenicline appeared to be similar that of dexamethasone based on their suppressive activities on pro-inflammatory cytokines. Furthermore, varenicline could be repurposed for the treatment of inflammatory diseases due to its potential to suppress PG synthesis.

## Data Availability

The raw data supporting this article will be made available by the authors upon request.
